# Povidone-iodine ear wash and oral cotrimoxazole for chronic suppurative otitis media in Australian aboriginal children: study protocol for factorial design randomised controlled trial

**DOI:** 10.1186/s40360-019-0322-x

**Published:** 2019-07-27

**Authors:** Christine Wigger, Amanda Jane Leach, Jemima Beissbarth, Victor Oguoma, Ruth Lennox, Sandra Nelson, Hemi Patel, Mark Chatfield, Kathy Currie, Harvey Coates, Keith Edwards, Heidi Smith-Vaughan, Kim Hare, Paul Torzillo, Steven Tong, Peter Morris

**Affiliations:** 1 Menzies School of Health Research, PO Box 41096, Casuarina, Darwin, NT 0812 Australia; 2 Top End Health Services, Darwin, Northern Territory Australia; 3 Royal Darwin Hospital, Darwin, Northern Territory Australia; 4 University of Queensland, Brisbane, Queensland Australia; 5 Northern Territory Department of Health, Darwin, Northern Territory Australia; 6 University of Western Australia, Perth, Western Australia Australia; 7 Royal Prince Alfred Hospital, Camperdown, New South Wales Australia; 8 Royal Melbourne Hospital, Melbourne, Victoria Australia; 9 Royal Darwin Hospital, and Menzies School of Health Research, Darwin, Northern Territory Australia

**Keywords:** Chronic suppurative otitis media, Indigenous, Aboriginal, Cotrimoxazole, Povidone-iodine, Children, Ciprofloxacin, Randomised controlled trial

## Abstract

**Background:**

Chronic suppurative otitis media (CSOM) is a significant health issue affecting Aboriginal Australians. Long-term hearing loss can cause communication problems, educational disadvantage, and social isolation. Current standard treatment for CSOM in our region is twice daily dry mopping of the pus from the ear canal followed by instillation of ciprofloxacin antibiotic ear drops for up to 16 weeks, or until the discharge resolves for a period of 3 days. The treatment is long, laborious and fails to resolve ear discharge in 70% of cases in remote communities. Bacterial pathogens also persist. Povidone-iodine ear wash is the preferred method of clearing ear discharge in Western Australia. However, evidence of its effectiveness is lacking. In systematic reviews, topical antibiotics (ciprofloxacin) have been shown to be more effective than oral antibiotics or topical antiseptics. Currently, it is unclear whether there are any benefits of combining these treatments.

**Methods:**

This protocol describes a 2 × 2 factorial randomised controlled trial of two different interventions (povidone-iodine ear wash and oral cotrimoxazole), given as adjunctive therapy to standard treatment for CSOM. 280 children, between 2 months and 17 years of age, Indigenous or non-Indigenous, living in participating Northern Territory (NT) communities are randomised to standard treatment (dry mopping and ciprofloxacin drops) plus one of two topical treatments (dilute povidone-iodine ear wash or no wash) and one of two oral medication treatments (16 weeks of cotrimoxazole or placebo).

**Discussion:**

Current treatment of CSOM in our region shows that eradication of bacterial pathogens from the middle ear space and dry ears is often not achieved. This trial will evaluate the efficacy of adjunctive treatments of antiseptic ear washes and oral antibiotics. Clinical, microbiological and hearing outcomes will be reported.

**Trial registration:**

This trial (ACTRN12614000234617) was registered with ANZCTR on 05 April 2014.

## Background

In 2013, remote Indigenous communities participating in ear and nasopharyngeal carriage surveillance found that around 50% of young children (mean age 13 months) had otitis media with effusion (OME), 37% had acute otitis media (AOM without perforation), and 12% had chronic suppurative otitis media (CSOM). [1] CSOM is one of the most significant health problems affecting young Aboriginal children in Australia today [2, 3] having compounding effects on hearing, behaviour and learning. It was not that long ago that children with pus draining from their ears were a common sight in remote communities with more than 50% of young children in some remote communities having long term CSOM. [4, 5] Although the rates of CSOM have decreased, the NT hearing service reported that 53% of young Aboriginal children living in remote NT communities still have some form of hearing loss. [6, 7] Of all forms of ear disease, children diagnosed with CSOM had the highest levels of hearing loss. It should be noted that children with dry perforations and wet perforations (or CSOM) may be interchangeable, depending on the season and the child’s upper respiratory infection status. Combined rates of wet and dry perforation between 2007 and 2011 were 26% (15 and 11%) in 0–5 year olds, 26% (10 and 16%) in 6–11 year olds, and 37% (12 and 25%) in 12+ year olds respectively [7]. While effective timely treatment of acute otitis media with perforation (AOMwiP) aims to prevent CSOM, the challenges involved in delivering appropriate treatment in remote settings mean that a large group of children develop chronic severe ear disease [8]. The early age of onset, the severity of the hearing loss, and the persistence of this infection, means that improving medical management for this condition is a priority. Once CSOM has become established, it is extremely difficult to treat. Ear discharge failed to resolve at the end of topical antibiotic therapy in 70% Indigenous children in our previous randomised trial. [9] While topical antibiotics have been shown to be more effective than oral antibiotics and topical antiseptics, it is unclear whether there are any benefits of combining these treatments [9–11]. Other treatment options, such as prolonged antibiotic intravenous treatment in hospital have not been used (or accepted) in the management of children from remote Aboriginal communities [12, 13]. Topical quinolone antibiotics (e.g. ciprofloxacin) are currently the recommended first line treatment for CSOM and are theoretically more active against the bacteria commonly associated with CSOM (*Pseudomonas aeruginosa*), but have failed to eradicate all pathogens involved (particularly *Staphylococcus aureus*). Our previous randomised trial comparing topical treatments, ciprofloxacin versus framycetin (0.5%), gramicidin, dexamethasone (sofradex®) for CSOM found that *Staphylococcus* species were the most commonly isolated pathogens in ear discharge after treatment (present in 75% of specimens). We also documented an as overgrowth of fungal species. [9] There are no large studies describing the microbiology of CSOM in Aboriginal children. There is also lack of evidence for the effectiveness of combining antiseptic ear washes (povidone-iodine) with topical antibiotics (ciprofloxacin or sofradex®). The only randomised controlled trial (RCT) in Aboriginal children to describe a treatment that was effective in the majority of children with CSOM (around 75%) used the combination of povidone-iodine ear washes followed by ciprofloxacin ear drops. However, there were important differences in the severity of disease in these children compared to those currently living in remote communities of the NT [14]. Based on the clinical trials literature, treatment with oral antibiotics is not recommended routinely for CSOM and are usually less effective than topical antibiotic treatment. In our most recent CSOM study, 75% of children who sought clinical care from their regular health care provider were prescribed amoxycillin. It is unclear whether this oral treatment is ever indicated. [15–17] The only published placebo-controlled trial found the addition of oral cotrimoxazole to topical aminoglycoside plus steroid treatment reduced persistent discharge by 25 and 15% after 6 and 12 weeks of treatment [18]. Currently, there is a preference for surgical repair of the tympanic membrane in older children with chronic perforations that are dry at time of surgery [19]. This RCT will determine the clinical and bacteriological efficacy of oral antibiotics and povidone-iodine ear washes in addition to current recommended treatment (dry mopping plus topical antibiotic drops). Outcomes after four months, and at 12 months follow-up will include degree of resolution of ear discharge, bacterial pathogen persistence, healing of the tympanic membrane and hearing levels.

## Methods

### Study design

This is a parallel group, 2 × 2 factorial design, randomised (allocation concealed), assessor blinded, controlled clinical trial. This RCT was conducted in the Northern Territory (NT) and aimed to compare current standard treatment (twice daily dry mopping, ciprofloxacin drops and weekly clinic review) with two adjunctive therapies. Each child was randomised to receive standard treatment plus one of two topical treatments (povidone-iodine ear wash or no ear wash) and one of two oral medication treatments (cotrimoxazole or placebo) for 16 weeks.

### Participants and setting

All children (Aboriginal and non-Aboriginal) aged 2 months to 17 years of age living in remote or urban NT communities with a tympanic membrane (TM) perforation and a diagnosis of CSOM in at least one ear were eligible. Children with the following were excluded: i) previously randomised in this study; ii) ciprofloxacin, cotrimoxazole or iodine allergy; iii) mastoid or tympanoplasty surgery in the preceding 12 months; iv) ear surgery scheduled in the next 4 months; v) congenital ear or hearing problems; vi) known immunodeficiency; or vii) pregnancy.

### Ethics, consent and permissions

This study was approved and reviewed six monthly by the Human Research Ethics Committee of the Northern Territory Department of Health and the Menzies School of Health Research (HREC-2014- 2170), and the Central Australian Human Research Ethics Committee (CAHREC-14-228). The trial was conducted in accordance with the principles of Good Clinical Practice (GCP), the Declaration of Helsinki and the National Health and Medical Research Council (NHMRC) recommendations for research in Indigenous communities. Each participating community council/board provided written approval of the study to the ethics committees. Freely given informed written consent was obtained in person, by the research nurses from every subject’s parent/guardian (plus assent from children over 10 years of age) prior to enrolment. The study information, provided face-to-face in written, oral, and pictorial formats have been approved by the Ethics Committees. An interpreter was used when requested (or deemed necessary by the researchers). Sufficient time was provided to ask questions, discuss participation with relevant others, and obtain further study details prior to signing and dating consent forms. Consent included permission for their child to have an ear examination, nasal and ear swabs, a general child health check at baseline, 4 and 12 months follow-up visits. Details of data collection at each of the scheduled assessment times are summarised in Table 1. Signed consent was also sought to access their child’s medical records, complete a lifestyle interview regarding information on likely risk or protective factors for otitis media, to use the study and standard medicines, and to be contacted by study researchers at the participant’s home and via phone or social media, for the duration of the clinical trial.

**Table 1:** Schedule of assessments

Visit #	0	1	2	3
Event	^a^Screening	Day1	16weeks	12months
Informed consent				
Check informed consent				
Randomisation^b^				
Ear exam				
Check inclusion/exclusion criteria				
Nose and ear swabs				
Lifestyle questionnaire				
Clinical note review				
Check recent audiology				
General health check				
Record antibiotic/study medication use				
SAE reporting^c^				

^a^Screening is an ear exam to assess ear status and can occur on Day 1

^b^ Randomisation may be conducted at the time of screening

^c^Severe adverse events reporting will occur throughout the study

### Randomisation

Eligible children whose parent or guardian provided signed informed consent were randomised to one of the four treatment options according to a computer-generated sequence. This was obtained by a phone call (interactive voice response system) to the NHMRC Clinical Trial Centre (CTC). Children were stratified according to community and age (<4y, 4-10y, >10y) using a minimisation procedure**.** The allocation sequence was concealed from all investigators throughout the study. Study staff were blinded to the oral component of the treatment at randomisation and follow-up assessments. However, it was not possible to blind the topical treatment groups. All laboratory staff are blinded. The randomisation of an individual participant would only be unblinded in emergency situations where the chief investigator and study’s medical monitor feels the emergency cannot be adequately addressed without knowing which treatment was delivered.

### Intervention

Intervention 1: The active oral medication used for this trial is cotrimoxazole suspension (or tablet for children over 12 years of age). A non-active formulation of the exact colour, taste and appearance as the intervention was used as the placebo. Cotrimoxazole suspension was given as 4 mg/kg per dose (of trimethoprim component) twice a day orally for 16 weeks. The placebo was administered in an identical manner.

Intervention 2: The topical treatment was aqueous povidone-iodine 10%, diluted in clean, room temperature water (1:20). Each discharging ear was syringed twice daily using a 20 ml syringe for up to 16 weeks or until the ear was dry for 14 days (confirmed on two clinical examinations 1 week apart by study or clinic staff). Treatment was recommenced if discharge becomes present again.

### Safety monitoring

Serious adverse and medically significant events including reactogenicity were regularly monitored. Serious local or systemic reactions were noted at the time of randomisation, on day three and regularly throughout the trial by reviewing all presentations to the clinic and hospital and asking standardised questions (in person or by phone call) to the families. All adverse events were reviewed by an independent data safety monitory board (iDSMB), and reported to the ethics committees. The iDSMB, its quorum and detailed study stopping rules were established prior to the commencement of the study and are included in the study protocol. No modifications to the protocol would be made without the approval of the chief investigator and communication with all investigators and other adjudicating bodies including the iDSMB, ethics committees and Indigenous reference group.

### Outcome measures

The primary outcome measure was the proportion of children with clinical failure after 16 weeks (4 months) of treatment. Clinical failure was determined by clinical examination for the presence of any ear discharge. Additional secondary outcome measures at 4 months and 12 months included: i) hearing level, determined by audiometry (performed by trained audiologists) and summarised as the pure tone 4 frequency average hearing level at 500, 1000, 2000 and 4000 Hz in the better and worse hearing ears; ii) failure to improve (determined by perforation size and amount of discharge); iii) change in perforation size and amount of discharge (determined by comparison of standardised assessment at baseline with end of study); iv) time to cessation of discharge (determined by review of health records); v) adherence to recommended management including attendance for specialist review (determined by review of health records); vi) presence of recognised bacterial pathogens in nose and ear discharge (determined by standard microbiological culture and antimicrobial sensitivity methods); vii) complications (defined as development of a related illness requiring additional medical treatment); and viii) side effects (defined as development of illness requiring cessation of prescribed treatments). Additional secondary outcomes at 4 months and 12 months from randomisation included the proportion of children with any carriage of pneumococci and any carriage of vaccine or non- vaccine serotype pneumococci and any carriage of NTHi. All children were followed up for any adverse events (AE) and serious adverse events (SAE) by reviewing the child’s clinic and hospital records and asking the parents about any side effects of the treatments between and at visits. We documented any contact the family or child had during the study with the clinic or hospital, including all relevant investigations and treatments. Regular, scheduled home visits, phone calls, SMS texts and social media contact with the families were made and recorded by study nurses or community researchers to support and promote participant retention; and monitor adherence.

### Sample size

We estimate that at least 70% of children receiving current standard therapy for CSOM will (unfortunately) not be cured after 16 weeks of treatment. [8] The target enrolment was 280 participants. This study would have 90% power to detect an improved outcome in an additional 20% of children for each intervention (at the 5% significance level), assuming 10% loss to follow up or withdrawal.

### Data and specimen management and storage

All data collected was recorded on standardised forms in participant workbooks. Researchers enter data electronically into a MySQL database with secured Microsoft Access front-end as soon as possible after collection. All data are securely stored in a locked facility and microbiological specimens are stored securely at low temperature (−70C) in preparation for processing. Carriage data are entered directly onto the MySQL database by the laboratory scientists. Missing data and logic checks are carried out on all data with 10% of electronic data carefully cross-checked with paper forms.

### Statistical methods

All participants randomised are eligible for inclusion using principles of intention- to-treat (ITT) analyses of outcomes. Participants with complete data who completed all study visits are eligible for inclusion in the as-per-protocol analyses (APP). Baseline characteristics will be reported according to the treatment groups randomised: (i) povidone-iodine wash plus cotrimoxazole; ii) povidone-iodine wash plus placebo; iii) no povidone-iodine wash plus cotrimoxazole; and iv) no povidone-iodine and placebo. Based on CONSORT recommendations, there will be no inferential statistics for the baseline characteristics. Continuous variables will be presented by means, standard deviations, medians and interquartile ranges as appropriate. Counts and percentages will be used to describe categorical variables. Groups of size *N* = 140 will be compared, i.e. children who were randomised to povidone-iodine ear wash will be compared to children who were not, and children who were randomised to cotrimoxazole will be compared to children who were randomised to placebo. An interaction test to provide evidence of no interaction between the two interventions will be carried out first. Subgroup analysis will be assessed using interaction terms in a binomial regression models for binary outcomes and linear regression model for continuous outcomes at 4 and 12 months. The subgroups will include sex, age, category of CSOM and community.

#### Primary clinical outcomes

The primary endpoint is the proportion of children with clinical failure of CSOM after 16 weeks (4 months) of treatment. A binomial regression with logarithmic and identity link will be used to estimate the main effects of the two interventions. Therefore, the relative risk and the risk difference, respectively, with their 95% confidence interval will be reported for the crude and adjusted estimates. The adjustment set will comprise of the stratification factors (age group and community) and the other intervention.

#### Secondary clinical outcomes

The proportion of clinical CSOM that fails to improve (determined by perforation size, amount of discharge, time to cessation of discharge) at 4 and 12 months will be analysed in the same manner as the primary clinical outcome described above.

#### Carriage outcomes

The proportion of children with carriage of any capsular pneumococci, carriage of vaccine serotype capsular pneumococci, non-vaccine serotype capsular pneumococci and any non-typeable *Haemophilus influenzae* (NTHi) *Pseudomonas aeruginosa, Streptococcus pyogenes, Streptococcus aureus, Proteus species* and coliforms at 16 weeks (4 months) and 12 months will be described. A binomial regression will be used to estimate the crude and adjusted effect of the two interventions on carriage outcomes in the same manner as earlier described for the primary clinical outcome.

#### Audiology outcomes

Differences in hearing level at 12 months will be assessed using a simple linear regression. The absolute mean difference and the 95% confidence interval between the two interventions will be reported. The baseline hearing level will be adjusted in an analysis of covariance (ANCOVA). Further, the stratification factors and the other intervention will be included as explanatory variables to assess their effects on the hearing outcome.

#### Other outcomes

Complications, defined as proportions of children that develop illness requiring additional medication and proportions of children with any illness requiring cessation of prescribed treatment will be compared between treatments using a Chi-square test.

#### Missing data

The number of participants with primary endpoint and other data available at required timepoints, will be reported. No data imputation for missing data will be carried out. Where reasonable, complete case analysis will apply. All analysis will be two-tailed and statistical significance will be set at *p* < 0.05, using Stata 15.1 (StataCorp, College Station, TX, USA).

## Discussion

Because of the persistent, chronic nature of CSOM, treatment remains challenging and arduous for clinicians and families. Current best practice treatment of CSOM is not very effective. This means that CSOM remains unacceptably common and severe in Aboriginal children. Improved treatments are necessary. This trial will evaluate the effectiveness of two adjunctive treatments in addition to standard treatment and report clinical, microbiological and hearing outcomes. The results of this trial will become the best available evidence to guide the medical treatment of Indigenous children with CSOM. Outcomes of this research will be disseminated to participants, their communities and all stakeholders in person (where possible) and by publication.

## Trial status

Ongoing. Recruitment commenced in 2015 and finished in December 2018. Some outstanding data collection continues and data cleaning has commenced.

## Data Availability

These is no minimal datasets or material presented in this manuscript that would be necessary to interpret. Data that supports this study protocol can be obtained from the corresponding author, providing appropriate ethical and community approvals are provided. Data are not available on a public repository.

## Abbreviations

AOMwiP: Acute otitis media with perforation
AOMwoP: Acute otitis media without perforation
APP: As-per-protocol
CSOM: Chronic suppurative otitis media
CTC: Clinical Trial Centre
DP: Dry perforation
GCP: Good Clinical Practice
iDSMB: Independent data safety monitory board
IPD: Invasive pneumococcal disease
ITT: Intention-to-treat
NHMRC: National Health and Medical Research Council
NT: Northern Territory
NTHi: Non-typeable *Haemophilus influenzae*
OM: Otitis media
OME: Otitis media with effusion
TMP: Tympanic membrane perforation
